# Theoretical Approaches and Practical Assessment of Socio-Economic Effects of Desertification in Mongolia

**DOI:** 10.3390/ijerph17114068

**Published:** 2020-06-07

**Authors:** Erdeni D. Sanzheev, Anna S. Mikheeva, Petr V. Osodoev, Valentin S. Batomunkuev, Arnold K. Tulokhonov

**Affiliations:** Baikal Institute of Nature Management of the Siberian Branch of Russian Academy of Sciences, 670047 Ulan-Ude, Russia; asmiheeva@binm.ru (A.S.M.); osodoev@binm.ru (P.V.O.); bvalentins@binm.ru (V.S.B.); aktulohonov@binm.ru (A.K.T.)

**Keywords:** desertification, socio-economic consequences of desertification, sociological survey, questionnaire survey, households

## Abstract

In this paper, we consider the effects of desertification in Mongolia, where the area of degraded land has increased significantly in the recent decade. Currently, almost the entire territory of the country is subject to varying degrees of degradation. The intensity of the desertification processes in different natural zones is influenced by both natural climatic and anthropogenic factors. The purpose of this study is to evaluate the impact of desertification on environmental and socio-economic living conditions, as well as on living standards of the local population. In this work, for the first time, the socio-economic aspects of desertification have been studied on a common methodological basis in different Mongolian aimags over a ten-year period. In order to carry out in-depth research, we used the submeridional and sublatitudinal principles for selecting the model study areas, as well as specific criteria and expert assessment. We used a sociological survey as the main method, based on a designed questionnaire, which was translated into Mongolian. The questionnaire includes questions regarding the influence of desertification on traditional nomadic farming, health of family members, water supply of households, water quality, living standards, etc. The results of the sociological surveys made it possible to draw conclusions on the impact of desertification on households, to identify the main problems of local people, and to describe the dynamics of the socio-economic status of the population living in the model areas. Our studies have demonstrated the intensification of the impact of desertification processes in different natural zones, administrative-territorial units and settlement systems in Mongolia.

## 1. Introduction

Desertification is one example of a serious environmental problem that poses a threat to certain countries, as do other global problems of mankind, such as climate change, biodiversity loss, environmental pollution, etc. [1,2]. This is why studying the reasons and effects of desertification is an important scientific task.

In terms of studying desertification processes, the Mongolian territory is of great interest, as it is located in different natural zones, extending from the Gobi Desert in the south to the taiga in the north. In different aimags, depending on the latitudinal location, desertification progresses with various stages of intensity, affecting the living standards of the population, as well as environmental and socio-economic living conditions [3]. Economic and geographic research will allow not only to reveal the features of the desertification processes in different natural zones of Mongolia, but also to compare the manifestation of social and economic problems caused by these processes.

Desertification refers to land degradation in arid, semi-arid, and dry sub-humid areas resulting from various factors, including climatic variations and human activities [4]. Researchers vary in their estimates of the extent of desertification in Mongolia. Some of them note “…overestimated pasture degradation rates; according to their estimation, 1.0–18.0% of pasture area can be classified as highly degraded, 25.0–40.0%—moderately degraded, 33.0–40.0%—slightly degraded” [5]. Mongolian researchers consider the land proportion, degraded due to desertification, to be about 70.0% [6]. It is shown in other studies that “…approximately 77.8% of the country is desertified, of which 16.7% are areas where the processes of land degradation have heavy and severe forms” [7].

According to the Ministry of Environment and Tourism of Mongolia, 76.8% of the Mongolian territory is exposed to desertification due to natural causes, irresponsible mining, and the misuse of pastures. Over the past 15 years, there has been an increase in desertification and land degradation processes. According to the above estimates, 49.0% of the country’s area has undergone desertification and land degradation due to inappropriate human activities, while 51.0% are associated with natural and climatic changes [8].

The major factor driving the desertification processes in Mongolian arid territories is the degradation of vegetation cover, as well as ongoing changes in the structures of plant communities due to climate change. “The degradation of vegetation cover occurs to a large extent as a result of overgrazing, increased load on pasturelands, and decline in the number of plant species” [9,10,11]. Given the global climate changes and progressive aridization that have taken place in recent decades, as well as the changes in traditional nomadic farming in Mongolia, we can assume that desertification has affected almost the entire territory of the country.

In order to characterize the mutual influence of aggravating environmental problems on socio-economic systems at different territorial levels, we conducted studies, united by the concept of “consequences of desertification”. It is often necessary to assess the consequences of a process or an action to meet scientific or practical needs (e.g., to justify investments to mitigate impacts, to evaluate the effectiveness of undertaken or planned activities, to calculate various compensation costs or payments). Hence, there is a need to develop reliable approaches for scientific and methodological assessments of the consequences. In our case, the necessity of research on the territory of Mongolia is determined by increased desertification, the migration of rural populations (primarily young people), an increase in poverty, the deterioration of skills of traditional nomadic farming, and identification of the main problems [12,13]. Previous studies demonstrated the dynamics of desertification, its physical and geographical characteristics, probable changes resulting from different pathways of the desertification process, and the current socio-economic status of a soum or an aimag [5,14].

The purpose of this study is to evaluate the impact of desertification on environmental and socio-economic living conditions, as well as on the living standards of the local population. In this work, for the first time, the socio-economic aspects of desertification have been studied on a common methodological basis in different geographical zones over a ten-year period.

Existing methods of research on the socio-economic consequences of desertification are based on estimates of macroeconomic indicators, the correlations between urbanization and natural processes, the comparative analysis of biological productivity of grassland in different natural zones, and the mapping of desertification processes [5,13,14]. The method of the sociological survey allows for obtaining unbiased data on the social and economic situation, the actual living conditions of mobil pastoralist in the areas that are affected by desertification.

## 2. Materials and Methods

### 2.1. Overview of the Study Area

**Figure 1:**
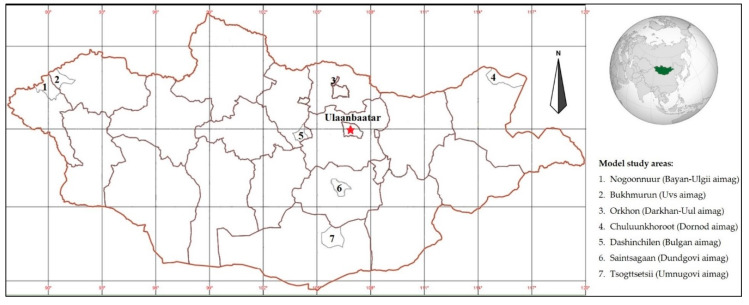
Overview map of the model study areas in Mongolia.

Mongolia is located in the central part of East Asia and has an area of 1.56 million km^2^ (Figure 1). It is a landlocked country bordered in the north by Russia and in the south by China. Most of the territory is occupied by a weakly dissected plateau, which lies 900–1500 m above sea level. In the north, west, and south-west are the main mountain systems and massifs (the Eastern Sayan, the Khentii, the Mongolian Altai, the Gobi Altai, etc.), divided by depressions and valleys. The Khangai mountain massif is in the central part of the country [15,16].

Mongolia is characterized by a sharply continental climate with harsh winters and dry hot summers. The amount of precipitation is 200–500 mm per year, and in the Gobi Desert areas, less than 100 mm per year [15,16]. The arid climate combined with anthropogenic activity are the main causes of desertification in Mongolia.

There is a river drainage system in the north of the country. The largest rivers are: the Selenga river with its tributary the Orkhon, as well as the Kherlen, the Onon, the Zavkhan, and the Khovd. There are only intermittent streams in the Gobi Desert. Most lakes are located in the west and northwest of the country [15,16].

The most common types of soils in Mongolia are: mountain steppe chestnut soils (over 60.0% of the country’s area), mountain steppe black earth soils, and brown soils with high salinity (mainly in the Gobi Desert). Grassland soils are typical for valleys and lake depressions. More than 80.0% of the country’s territory is occupied by the vegetation of mountain steppes, dry plain steppes, and desert steppes. Forests occupy about 10.0% of Mongolian territory. The rich diversity of wildlife in Mongolia encompasses 138 species of mammals, 274 species of birds, eight species of amphibians, 22 species of reptiles, and 75 species of fish [17].

In 2018, the total population of Mongolia was 3.2 million people [18]. The average population density was 2.1 people per km^2^. The central and northern parts of the country are the most densely populated. The modern settlement structure has been influenced by natural and economic factors. The proportion of the urban population was 67.4%.

Mongolia is a transition economy which uses market-based methods. According to official data from 2018 [18], the total GDP in comparable prices was USD 13,107.8 million, the GDP per capita was about USD 4000. In the structure of GDP, the services sector accounted for 46.3%, mining—24.6%, industry—10.9%, agriculture and forestry—10.7%, construction—4.3%, and other sectors—3.2%. The private sector share of GDP was 77.1%. The main sources of revenue to the state budget are exports of mining products, agricultural raw materials, and the products of their processing.

### 2.2. Justification for Selection of Model Study Areas

The desertification of the Mongolian territory is one of the key problems, since most of the territory is subject to a varying degree of aridization and desertification processes. A review of publications in this field revealed that previous studies [6,7,19,20,21,22] were mainly devoted to identifying the causes of desertification, examining the dynamics of aridization and desertification using GIS technologies and remote sensing data, and developing methods to combat desertification.

The literature review revealed that almost no research has been conducted with respect to theoretical and methodological approaches to studying the socio-economic effects of desertification. The complexity of developing methodological approaches is related, not only to the absence of theoretical developments, but also due to the multifaceted nature of the subject of study. The socio-economic aspects of desertification, despite their importance for local people, remained beyond the scope of scientists and experts.

There are a number of papers devoted to this topic [23,24,25]. These papers describe developed methodological approaches to assessing the consequences of desertification, its environmental–economic effects and socio-economic damage.

In order to study the impact of desertification on the economy and population, it is necessary to make a representative selection of areas of model study with typical characteristics, taking into account the impossibility of conducting continuous surveys, as well as vast desert areas and the diversity of their natural and socio-economic characteristics.

We therefore used the submeridional and sublatitudinal principles for selecting the model study areas. In the submeridional direction, we selected the areas that are located in different climatic and natural conditions. In the sublatitudinal direction, the model areas are located in the northwest and northeast of the country at the border between Mongolia and Russia.

The selection of model areas was carried out on the basis of common criteria and expert assessment. The following indicators were used as criteria: representativeness of the area; desertification rate; environmental situation; socioeconomic level of a soum; economic specialization—animal husbandry; increase in the number of livestock; population migration. Scientists from the Institute of Geography and Geoecology of the Mongolian Academy of Sciences (IGG MAS), the Institute of Economics of the Mongolian Academy of Sciences, and Mongolian State University, along with specialists from ministries and departments, acted as experts. The administrations of aimags and soums and chiefs of bags (administrative units in Mongolia) provided substantial assistance in organizing field studies and sociological surveys.

Seven soums were then selected using expert input, the analysis of particular transformations of natural complexes, and long-standing data of socio-economic dynamics, where in-depth field studies and sociological surveys were carried out in 2009–2012 and 2018–2019 (Figure 1). We compared our new findings against the previous data, showing the desertification impact on the socio-economic situation of the population, and revealed regional characteristics.

The justification for the selection of the model areas was made on physical, geographical, and socio-economic indicators. The model areas are located in various aimags of Mongolia, located in different natural zones. The selected model areas in Mongolia are characterized by low and medium levels of economic development.

The first model area (Dashinchilen soum) is located in Bulgan aimag. The area of the soum is 2300 km^2^. It is located in the basin of the Orkhon and the Tuul river basins, near the offsets of Khangai–Khentii ranges in the steppe and the forest steppe zones. The main water course is the Khar Bukhyn River [15,16]. The soum has a population of 3000 people. The population density is 1.3 people per km^2^, which is below the national average [18]. The breeding of livestock, mainly sheep and goats, is a primary economic activity of the local people in this soum.

The second model area (Saintsagaan soum) is located in the north-eastern part of Dundgovi aimag. The area of the soum is 3400 km^2^. Its territory is located in the steppe and dry steppe zones, partially in the Gobi Desert. The soum is predominantly characterized by a flat relief, with mountains severely destroyed by weathering processes. There are practically no permanent water courses in the territory of the soum [15,16]. As of 2018, the population of Saintsagaan was 15,800 people. The population density was more than twice the national average of 4.6 people per km^2^ [18]. Saintsagaan soum lies in the center of the aimag and combines urban and rural areas. The main economic activity of the local people is animal husbandry. The industry is undeveloped. Located 32 km from Mandalgovi is the Tevshiin Gobi coal mine, where an average of 70,000 tons of coal are extracted annually. 

The third model area (Orkhon soum) is a part of the small Darkhan-Uul aimag. The area of the soum is 477.87 km^2^. It is located in the valley of the Orkhon River in steppe and forest steppe zones with flat relief and minimal altitude differences [15,16]. The presence of permanent water courses encourages the local population of the soum to develop crop production. In 2018, the population was 3300 people. It is one of the most densely populated areas in the country (6.8 people per km^2^) [18]. The main industries are animal husbandry and irrigated crop production.

The fourth model area (Tsogttsetsii soum) is in Omnogovi aimag, in the south of Mongolia and in the north of the Gobi Desert. The area of the soum is 7200 km^2^. Its territory is characterized mainly by flat relief with no surface water bodies [15,16]. As of 2018, the population of Tsogttsetsii soum was 8000 people. The population density here is almost twice as low as the Mongolian average and amounted to 1.1 people per km^2^ [18]. In Tsogttsetsii, the main economic sectors are currently mining and tourism. Crop production is not developed here because of unfavorable natural and climatic conditions.

The fifth model area (Chuluunkhoroot soum) is located in Dornod aimag in the north-east of the country. The area of the soum is 6500 km^2^. The soum is in the dry steppe zone and is characterized by steppe vegetation. Its relief is represented by shallow depressions and low mountains. The main surface water bodies are the Uldz River, the Imalka River and Lake Barun-Torey [15,16]. In 2018, the population was 1900 people [18]. Due to the low population density (only 0.3 people per km^2^), farming areas are scattered across the territory. Farming (mainly livestock breeding) is the primary economic activity of local people.

The sixth model area (Nogonnur soum) is in Bayan-Ulgii aimag, in the north-west of Mongolia. The area of the soum is 5200 km^2^. Nogonnur is located in the foothills of the Mongolian Altai and is characterized by sparse high-mountain vegetation. The Khovd and the Bukhmuren rivers, as well as their tributaries, flow through the territory of the soum. Lake Achit is to the east of the soum [15,16]. In 2018, the population was 8000 people [18]. The population density is lower than the Mongolian average and amounted to 1.5 people per km^2^. Nomadic farmers use a transhumant grazing system here. A free economic zone entitled “Nogoonnuur” has been established in the territory of the soum.

The seventh model area (Bukhmuren soum) is located in Uvs aimag on the border with Bayan-Ulgii aimag. The area of the soum is 3700 km^2^. Bukhmuren is located on the Gatsuurtai, the Yamaat, and the Bairam mountain ranges. The Bukhmuren River runs through the territory of the soum, flowing into Lake Achit [15,16]. In 2018, the population size was small—1200 people [18]. The population density is also low—0.6 people per km^2^. The economy of the soum is mainly based on animal husbandry with the use of the transhumant grazing system.

### 2.3. Review of Methodological Approaches

The importance of research on the development of the approaches to studying the natural and anthropogenic effects in desertification zones arises from: ensuring a safe environment for the population, increased desertification, and the need for effective monitoring as an instrument for the prevention of hunger and poverty through political and economic measures [26,27].

Many geographers, economists, and landscape scientists described the spatio-temporal dynamics of the natural and anthropogenic effects on various environments of Mongolia, and noted the intensification of the processes and the complexity of their influences on various sectors of the economy [28,29,30].

The modern scientific assessment of various effects on the natural environment of residential areas, as well as the prediction of changes in the substance dynamics, have been developed in later studies [31,32]. Any transformation of a natural landscape leads to the changing of the whole natural complex, which in turn negatively affects the territorial organization of nature, society, and the economy [33].

The consequences of desertification include environmental, economic, and social losses [34]. Such losses include the following: the degradation of human capital associated with the decline in public health, forced migration and unemployment; the loss of the productivity of natural and man-made systems; environmental servicing irregularities; losses of environmental quality and comfort; environmental service expenditures; the costs of preventing ecosystem degradation; mitigation costs [35].

Some researchers note that there has been a growing awareness of the link between desertification and economic loss, especially for the poor, whose lives depend on the use of local natural resources, and desertification further increases poverty [36,37]. This leads to a mass migration of people, so the effect of desertification on this process is cumulative [38].

According to studies by [39,40,41], public opinion surveys can shape the development of national environmental policies, mechanisms for the preservation of landscapes, and the implementation of projects and programs. The identification of the most degraded agricultural areas using GIS tools allowed the selection of respondents for surveys among local farmers [42].

The sociological methods have been applied for the characterization of the impact of desertification to various regions of the world [43,44,45]. Thus, the first national study of soil degradation in Iceland [46] was associated with public opinion surveys. According to the results of the surveys, most Icelanders recognized soil erosion as a serious environmental problem. The results of the surveys have become the basis for a new soil conservation program.

The method of the sociological survey has been successfully applied to desertification processes in studies by Russian [47,48], Chinese [49,50], and other researchers [12]. Furthermore, an analysis and comparison of the survey data with the data from other sources indicates the reliability of the results obtained using the sociological survey method.

**Figure 2:**
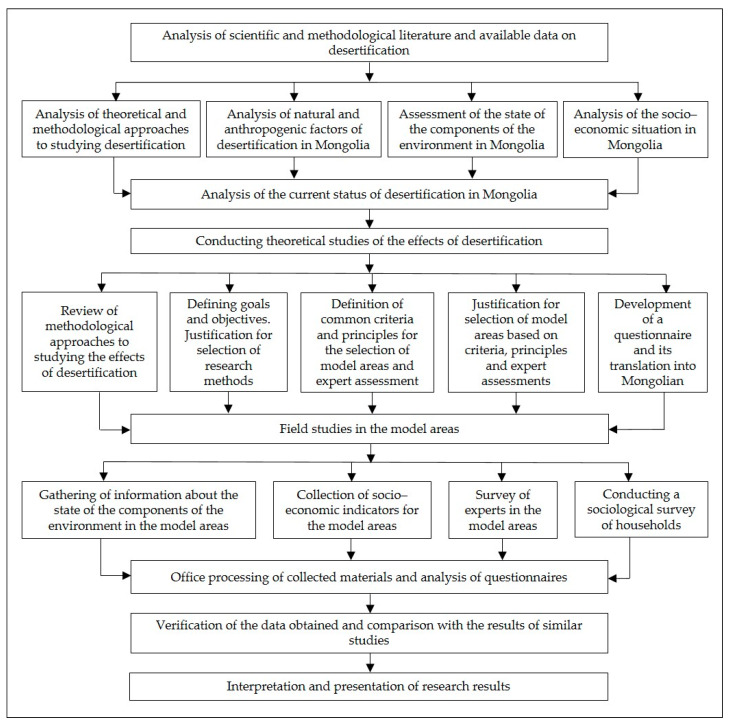
Research methodology.

The structure and content of the study was determined by a developed concept (based on the analysis and generalization of previously gained knowledge and facts about the studied process and objects and the identification of problematic situations) (Figure 2).

The developed conceptual approaches were based on the following structure:

- An analysis of theoretical approaches to assess the desertification process in Mongolia and the identification and characterization of natural and anthropogenic factors affecting the desertification process;
- A condition of components of the natural environment, the main sources of pollution, and the sectoral and territorial structure of the economy;
- The selection of the model study areas was justified on the basis of common criteria and expert assessment. The following indicators were used as criteria: representativeness of the area; desertification rate; environmental situation; socio-economic level of a soum; economic specialization—animal husbandry; increase in the number of livestock; population migration;
- The geographical principle was based on the submeridional and sublatitudinal principles for selecting the model study areas, which were located in different climatic and natural conditions;
- In our studies we used a sociological survey as the main method, well suited for the identification of natural and socio-economic effects, not only on households, but also on the social well-being, living standards, and health of the respondents and their families. The structure of the questionnaire was designed to characterize the dynamics of population income, public health status, and natural and economic conditions of life;
- Comparative analysis of the survey results.

A research group from the Baikal Institute of Nature Management of the Siberian Branch of the Russian Academy of Sciences (BINM SB RAS) designed the questionnaire to characterize the population income profiles and health status under the influence of natural and anthropogenic factors. It was developed in Russian and then translated into Mongolian, incorporating the comments made by scientists from the IGG MAS and Mongolian State University. When choosing representative households for the survey, the opinions of chiefs of bags and specialists of soum administrations were taken into account.

The questionnaire includes 30 questions grouped in sections, as follows: soum development issues; the financial situation of respondents and the reasons for its change; health status; the effect of desertification on the health of family members and on the management of nomadic households in general; the effect of water quality on the health of respondents, etc. The researchers from BINM SB RAS who speak the Mongolian language, as well as ones from IGG MAS and Mongolian State University, carried out the sociological surveys.

When conducting sociological surveys, population distribution patterns, the national mentality, and the customs and traditions of the Mongolian people were taken into account. The low population density in Mongolia and nomadic type of household management determine the spatial dispersion of the population. Nomadic households are one or two yurts (nomad’s tent) in which one family lives. The distance between neighbors can reach 30–35 km or even more. According to Mongolian hospitality traditions, a host (most often a man, the head of a nomadic household) always receives his guests and has a conversation with them.

The observation of the hospitality customs increases the surveying time spent on a household and the overall duration of field studies. The long distances between neighbor households leads to spending a great deal of time and money. At the same time, the largest distance between the model areas in the sublatitudinal direction was more than 2500 km, and in the submeridional direction, more than 800 km.

## 3. Results

### 3.1. Analysis of the Reasons for the Intensification of Desertification Processes in Mongolia

Desertification processes result from the integral impact of several negative factors [51]. The main causes of land degradation are, on the one hand, temperature and precipitation changes, and on the other hand, anthropogenic factors—pasture degradation due to a significant increase in livestock and changes in herd structure [6,19,52]. 

Researchers, who deal with forecasting climate change in Mongolia [53,54,55], assume that by the end of this century (2080), the air temperature in winter will increase by 3.4 degrees compared to the climatic norm of the period of 1961–1990; snowfall will increase by 13.9 mm, and rainfall in the summer will increase by 23.9 mm.

According to data from 48 weather stations in Mongolia, registered from 1940 to 2018, the ambient temperature has increased by 2.26 °C [56]. Therefore, further climate aridization is expected to occur in these climatic conditions, which may even slightly increase, but is still typical for this continental region of Asia [57]. The ongoing climatic changes in the territory of Mongolia have coincided in time with the transformation of sectoral and territorial structures of the country’s economy. The highest risk of desertification is associated with the following primary, natural resource-based sectors: agriculture, animal husbandry, mining (including open pit mining), and forestry (timber harvesting). 

To date, animal husbandry has been the basis of the Mongolian economy, contributing an average of 10% of the GDP [58]. Animal husbandry was formed under the influence of a unique centuries-old culture of nomadic pasture use, allowing the potential of natural resources to be preserved. At the same time [59], some have noted the negative changes in the state of the natural environment in Mongolia (in particular, biological resources), which have taken place against the backdrop of rapid urbanization and climate aridization. On the one hand, environmental protection has been declared a priority, and yet on the other hand, obsolete farming methods, the inefficient and inadequate use of financial resources, and the distribution of investments lead to contradictions between the environmental and socio-economic interests of society [60].

Non-rational livestock husbandry in Mongolia has the following causes: an increase in the number of livestock, changes in the sectoral and territorial structures of the nomadic economy, and a reduction in the traditional autumn–winter and summer nomadic movements of livestock breeders. The livestock population in Mongolia increased almost 2.6 times from 1990 to 2018 (Table 1).

Significant changes have taken place in the livestock structure due to market conditions, specifically an increase in demand for goat hair and cashmere products. In 1990, goats accounted for 19.8% of the total livestock population, and in 2018 40.8%, so the share of goats more than doubled. There is a decrease in shares of other livestock types (Table 1).

In addition to increasing the number of livestock and structural changes in animal husbandry, the traditional way of life of nomads (arats) has changed. There was a transition from four-season to two-season nomadic movements, resulting in the placement of cattle ranchers in separate pastures for a longer period of time. According to experts from the Institute of Geography and Geoecology of the Mongolian Academy of Sciences, labor costs expended for the production of one unit of agricultural output in nomadic households have increased by 20.0–25.0% over the past ten years.

In the opinion of B.O. Gomboev, the traditional way of life of animal husbandry was also influenced by “...modification of the administrative boundaries of soums, resulting in limiting the distances between nomads; the ongoing reassignment of land for other types of land use (including for military purposes); long-term lease of land to livestock breeders; replacement of local sheep breeds with astrakhan sheep” [62]. It should be noted that since the beginning of the 2000s, the areas of land reassigned for mining purposes have increased, the construction of new facilities has intensified, and cultivated areas, which are being developed mostly at the expense of grazing areas, have expanded.

Experts consider these changes as causes of conflict between livestock breeders, due to the lack of pastures, and the conflicts most often occur between locals and migrants. A.D. Gombozhapov writes that “land conflicts in the traditional places of nomadic farming become a common occurrence” [63]. The reasons for these increased tensions are the Mongolian government’s support for the development of crop production and the expansion of croplands and the transformation of land relations associated with changes in land legislation.

Changes in the herd structure and the concentration of livestock in a limited area near water supply sources over a long period of time have led to negative changes in the state of the natural ecosystems of pastures. This has resulted in an increased pressure on pastures, overgrazing, changes in the composition of plant species, and the destruction of vegetation, which leads to the accelerated degradation of pastures.

### 3.2. Key Indicators of Opinion Polls on Model Territories of Mongolia

A representative sampling was made in the model areas, taking into account the age and gender of the respondents. 

Sociological surveys of the Mongolian population were carried out in 2009–2012 in the following soums: Dashinchilen (Bulgan aimag), Saintsagaan (Dundgovi aimag), Orkhon (Darkhan-Uul aimag), and Tsogttsetsii (Umnugovi aimag) (Table 2). 

In 2018–2019, we carried out studies in the Mongolian border areas, and the findings point to similarities in the socio-economic problems of areas that are located at distances of up to 2500 km apart in different natural and climatic conditions. 

We selected three soums as the study subjects: Chuluunkhoroot (Dornod aimag), Nogoonnuur (aimag Bayan-Ulgii), and Bukhmurun (Uvs aimag) (Table 2). In Chuluunkhoroot soum, the sample proportion was 19.0%. In the other remaining soums, we carried out only a survey of experts, and the sample proportion varied from 0.5 to 3.0%.

### 3.3. The Desertification Influence on Traditional Farming and Public Health

The sample proportion ranged from 2.9 to 13.9%, ensuring the reliability of the results. Surveys were conducted on households in the first three soums, and of experts in Tsogttsetsii soum. In particular, specialists from the administrations of the soum and Umnugovi aimag were surveyed, along with experts from medical institutions and enterprises, who have a fairly complete understanding of the situation in the soum.

**Figure 3:**
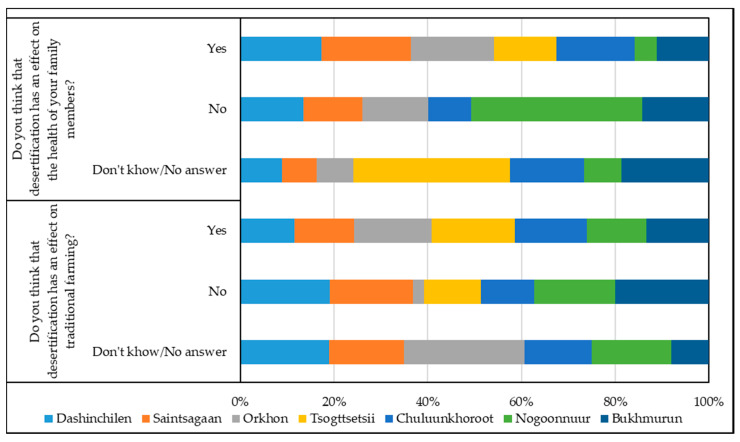
Distribution of answers to questions about the impact of desertification on traditional farming and public health.

In the questionnaire, the most important question is related to the desertification effect on traditional farming (Figure 3). 

In our opinion, the answer to this question helps to identify the local population’s opinion regarding the increase in desertification and its effect on their living and farming conditions. The majority of respondents (from 52.3% to 80.0%) answered this question in the affirmative. The further south the model area was, the more respondents confirmed the desertification influence on farming. About 30.0% of respondents in Dashinchilen and Saintsagaan soums believe that desertification does not affect farming. In Tsogttsetsii soum, which is located in the north of the Gobi Desert, 20.0% of the population do not associate the increase in desertification with the decline in nomadic farming.

The model areas we studied in 2018–2019 are located in the northwest and northeast of Mongolia in more favorable climatic and natural conditions. More than half of the respondents confirmed that desertification has an effect on traditional farming (Figure 3).

In our opinion, the assessment of the effect of desertification on human health is crucial for studying the impact of the environment on mankind. More than 50.0% of respondents (from 52.3 to 57.1%) in the model areas believe that desertification has an effect on the health of their family members (Figure 3).

About 1/3 of the respondents (from 29.5% to 32.9%) do not associate the decline in the health of their family members with an increase in desertification. The opinions of the respondents from each of the three model areas almost coincide. In Tsogttsetsii soum, the majority of experts (60.0%) found it difficult to answer this question during the survey.

The opinions of the respondents on whether desertification has had an effect on the health of their family members vary considerably in 2018–2019 (Figure 3). The following proportions of respondents consider that desertification affects the health of their family members: in Chuluunkhoroot soum—50% and in Bukhmurun soum—33.3%. At the same time, 87.5% of people surveyed in Nogoonnuur soum do not associate desertification with the health of their family members. On the basis of these answers, we suggest that the processes of desertification dominate in the east of the country and gives a foundation for respondents to connect its increase with the decline in health of the members of their families.

### 3.4. Household Water Supply and Water Quality

**Figure 4:**
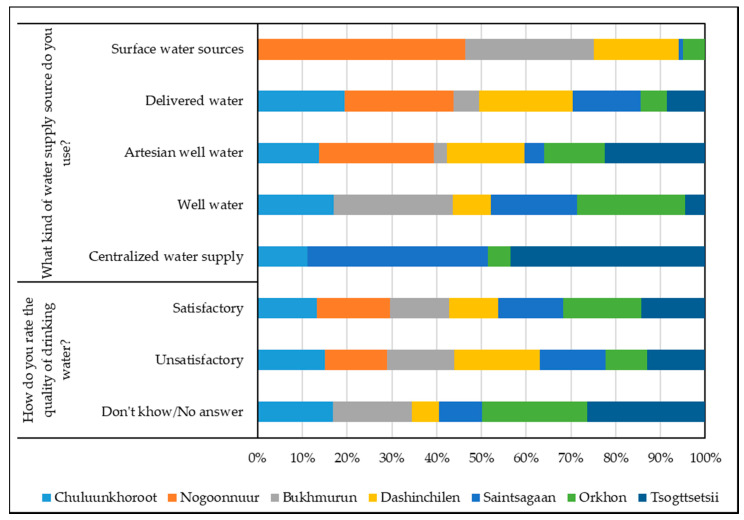
Distribution of answers to questions about water supply and water quality.

The availability of water supply sources remains the key issue for Mongolian households. This is of primary concern for animal husbandry, just as for crop production. According to our survey results, there are significant inequalities in the water supply of households in soums, which are located in different natural zones (Figure 4).

Ordinary wells account for almost half of all types of water sources in Orkhon soum (54.7%) and Saintsagaan soum (43.7%). In Dashinchilen and Orkhon soums, artesian wells account for more than 33.0% of all types of water sources and in Tsogttsetsii soum—60.0%. About 1/5 of respondents use delivered water in Dashinchilen and Saintsagaan soums. More than 1/4 of all respondents in Saintsagaan soum use a centralized water supply, along with 30.0% in Tsogttsetsii soum. Only in Dashinchilen soum do 18.0% of inhabitants use surface water sources. There are either no water sources or water is not suitable for use in other soums. It should be noted that only a small percentage of respondents are able to use several types of water supply sources.

According to the respondents, there is not enough of a water supply even though the available water is used very sparingly. In Orkhon, Saintsagaan, and Tsogttsetsii soums, 80.0–90.0% of respondents report the sufficiency of water for household needs, but in Dashinchilen soum—63.6%.

As expected, in 2018–2019, the water supply situation in model areas is better than in soums adjacent to the Gobi Desert (Figure 3). In Chuluunkhoroot soum, the population mainly uses ordinary wells (38.5%), artesian wells (30.8%), and delivered water (23.0%). In Nogoonnuur and Bukhmurun soums, local people consume water from surface water bodies (42.9% and 26.7%). In Nogoonnuur soum, inhabitants predominantly use water from artesian wells—57.1%; the percentage of delivered water users is 28.6%. In Bukhmurun soum, 60.0% of respondents use wells, and only in Chuluunkhoroot soum do 7.7% of respondents have access to a centralized water supply.

The quality of consumed water is an acute issue (Figure 4). Drinking water is of better quality in Orkhon soum, where 61.3% of locals are satisfied with its quality. In Saintsagaan and Tsogttsetsii soums, half of the respondents are satisfied with the quality of water (50.8% and 50.0%, respectively). Water of inferior quality is in Dashinchilen soum, with only 38.6% of its inhabitants being satisfied with its quality. The locals of Dashinchilen and Saintsagaan soums named salinity as the main drawback of the water consumed (83.3% and 79.2%, respectively). Respondents in Dashinchilen (16.7%), Saintsagaan (20.8%), and Tsogttsetsii (30.0%) soums named the hardness of water and high iron content as among the main drawbacks of their water quality. In Tsogttsetsii soum, people noticed a high mineralization of water. In Orkhon soum, 42.1% of inhabitants noticed the salinity of water, and 31.6% called the water dirty, muddy, and unsuitable for drinking.

In 2018–2019, in relation to the quality and availability of the drinking water supply, the respondents’ opinions are divided (Figure 4). In Chuluunkhoroot, Bukhmurun, and Nogoonnuur soums, approximately the same proportions of inhabitants describe the water quality as either “satisfactory” or “unsatisfactory”. The people surveyed in Bukhmurun soum mention shallow wells as being the main cause of poor water quality. In Chuluunkhoroot soum, the respondents described the drinking water as being saline and containing high levels of calcium and iron, and also noted the absence of water treatment, the pollution of water sources caused by gold mining, and the drying of springs.

### 3.5. The Socio-Economic Situation of Households

One of the questionnaire sections is designed to reflect the socio-economic situation of the respondents and identify income level groups. The majority of the local population notices negative trends, namely that earnings are barely enough to feed families and buy basic necessities. In some families, their income is not enough, even for food. In the absence of other earning opportunities (mainly due to unemployment), it drastically reduces the living standards of the population.

**Figure 5:**
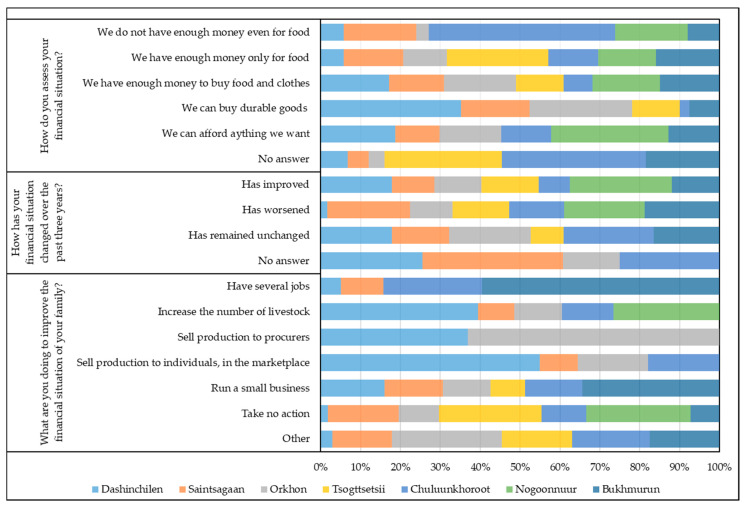
Distribution of answers to questions about the household’s financial situation.

We allowed the respondents to assess their financial situation (Figure 5).

In Orkhon and Dashinchilen soums, 21.5% and 29.5% of respondents, respectively, say they have enough money to buy durable goods, and more than 40.0% have enough to buy food and clothes. However, in Orkhon and Saintsagaan soums, 21.5% and 29.4% of the respondents, respectively, say they have enough money only for food and in Tsogttsetsii soum, 50.0%. The most difficult situation is in Saintsagaan soum—14.3% of respondents admit that the money they earn is not enough even for food.

In Tsogttsetsii soum, we mainly surveyed experts whose income level is higher than the average. However, in Umnugovi aimag, which Tsogttsetsii soum belongs to, retail prices for food and industrial goods are much higher than in the central and northern aimags, due to high transportation costs. Despite the fact that the income level is higher than the average, 50.0% of people surveyed in Tsogttsetsii soum consider the money they earn sufficient only for food, another 30.0% for food and clothes, and only 10.0% can buy durable goods (Figure 5).

As for the income level groups of the population in 2018–2019, 42.9% of rural inhabitants surveyed in Nogoonnuur soum say that they have enough money to buy food and clothes and 37.5% in Bukhmurun soum (Figure 5). In Nogoonnuur soum, 14.3% of respondents report that they can afford everything they want. This is mainly caused by the high-paying employment opportunities of the free economic zone “Nogoonnuur” in the soum. Slightly more than 6.0% of respondents belong to this group in Chuluunkhoroot and Bukhmurun soums.

In general, the social well-being in soums located in the north-west of Mongolia is much better than in the north-east of the country. Thus, 36.7% of respondents in Chuluunkhoroot soum report that they do not have enough money even for food and 24.5% have enough money only for food. In other words, more than 60.0% of the population is in a difficult economic situation. The people in other soums are also not in a good economic situation. 

The average monthly income of households, in comparison with the national and regional averages (Table 3), allows us to estimate income level in the model areas. The most favorable situation is in Dashinchilen soum, where the income level is quite comparable with the regional average, but less than the national average, by about 20.0%. In Orkhon soum, the income level is less than the average and amounts to approximately 90.0%. In Saintsagaan soum, the income level is only about 70.0% of the national and regional averages. 

In 2018–2019, the average monthly income of households is 13.0–20.0% lower than the Mongolian average (Table 3), so there is a decline in the standard of living compared to the national average.

Changing the financial situation of the respondents is one of the key issues which can reflect socio-economic dynamics. The percentage of respondents in different soums is as follows: those who consider that their financial situation over the past three years has improved—from 20.0 to 49.5%; those who say it has remained unchanged—from 20.0 to 49.5%, and those who say it has worsened—from 15.2 to 29.5% (Figure 5). At first glance, the most favorable situation is in Dashinchilen soum, where 2.3% of respondents consider that their financial situation has worsened. It should be noted that surveys in model areas were carried out at different times, making it possible to obtain comparable data that reflect environmental risks. In particular, a survey in Dashinchilen soum was conducted in 2009, prior to the dzud of 2010, when more than 1/3 of the total number of livestock died all over the country, which negatively affected the living standards of livestock breeders [3].

“Dzud” or “zud” (in Mongolian—“зуд”), or “lack of fodder,” is a natural disaster in animal husbandry areas (primarily in Mongolia), in which livestock are unable to find fodder under snow cover, and a large number of animals die from hunger and cold [65]. Saintsagaan soum, located to the south, was particularly affected by the dzud, which is clearly seen from the survey results (Figure 5).

The financial situation of the respondents has improved over the past three years: in Nogoonnuur soum—71.4% of respondents (Figure 5), in Chuluunkhoroot soum—21.7%, and in Bukhmurun soum—33.3%. Most respondents report that their financial situation has remained unchanged.

Among the main causes of decline in their financial situation, local people mention the rise in prices and inflation (37.5–50.0% of respondents), and loss of work—12.5–50.0%. Respondents in Chuluunkhoroot and Bukhmurun soums also consider low wages as a cause of the decline (20.5% and 25.0%, respectively).

Since household incomes are not sufficient to supplement family budgets, the population is forced to seek other income opportunities (Figure 5). In Dashinchilen soum, 42.4% of respondents have increased the number of livestock and 25.4% sell produce to individuals and in the marketplace. The shortage of pasture there is not yet as acute as in other soums, and there is an opportunity to increase the number of livestock. The same can be said about Orkhon soum, where 12.8% of the respondents expressed a desire to keep more livestock. A part of the soum population tries to run small businesses—from 10.0% in Tsogttsetsii soum to 18.6% in Dashinchilen soum.

The population of Orkhon, Saintsagaan, and Tsogttsetsii soums is trying to engage in vegetable growing to meet the increasing demand for vegetables. According to the survey, this is a recent trend to develop households and it is more common in Orkhon soum, which has more favorable natural and climatic conditions and sufficient fresh water for irrigation. The Orkhon River flows through the territory of this soum, having the greatest length (1124 km) and catchment area (132,000 km^2^) [66]. The river is one of the main tributaries of the Selenga River in Mongolia, and is an important source for domestic water supplies and irrigated farming.

In Chuluunkhoroot and Bukhmurun soums, low wages were also noted as the main reason for the decline in income (20.5% and 25.0%, respectively). In these soums, 12.0–13.0% of respondents report a drastic decrease in household income.

The possibilities of additional earnings are very limited due to natural, climatic, and socio-economic conditions in Mongolia, which only aggravate the inactivity of local people in search of financial opportunities. 

In Chuluunkhoroot soum, 30.6% of respondents take no action and in Bukhmurun soum—20.0% (Figure 5). Forty percent of respondents run small businesses in Bukhmurun soum and 16.7% in Chuluunkhoroot soum. At the same time, some residents work more than one job—20.0% and 8.3%, respectively. Only in Chuluunkhoroot soum do 8.3% of respondents indicate that they sell produce to individuals and in the marketplace. In Chuluunkhoroot and Nogoonnuur soums, 13.9% and 28.6% of respondents consider an increase in the number of livestock as a way to help solve their financial problems.

In contrast with the population of Orkhon, Saintsagaan, and Tsogttsetsii soums, the respondents here are less likely to engage in crop production, despite the favorable natural and climatic conditions for growing vegetables. There are more respondents in eastern and western soums who work more than one job to keep a family. This suggests more favorable employment conditions here. Additionally, there are more respondents running small businesses in Bukhmurun and Chuluunkhoroot soums.

Consequently, the financial situation of the respondents in the model areas is, in most cases, worse than the regional and national average levels. The households are highly dependent on external factors, which determine not only the size of the households, but also the characteristics and structure of the herds. Purchase prices for agricultural products encourage the population to increase the number of goats and sheep. This breaks the optimal balance of herds, which is the result of thousands of years of experience in traditional nomadic farming, and negatively affects the quality of pastures. Additionally, the number of livestock is largely determined by the availability of pastures and access to watering places. Some natural phenomena, such as dzud, can cause the death of livestock and thereby ruin households. The death of livestock as a result of dzud may force locals to leave their places and migrate to cities in search of work.

## 4. Discussion

Nowadays, there has been a lack of consensus on the amount of land affected by desertification in Mongolia and its proportion of the national territory. 

By various estimates, the proportion of land area affected ranges from 60.0% to 80.0% [5,6,7]. The main cause of modern desertification in Mongolia is the influence of both natural climatic and anthropogenic factors on natural landscapes [6,19]. Climate aridization and increased desertification are the main causes of negative changes in the environment, which affect the living conditions of the population.

Within the framework of the “Sustaining Interdisciplinary Collaboration Across Continents and Cultures: Lessons from the Mongolian Rangelands and Resilience Project” the livestock breeders in Mongolia were surveyed in order to assess the climatic and socio-economic changes and their impact on environmental and social living conditions [67].

In the works by the authors of [12,13], the main results of the sociological surveys are: the confirmation of data on the degradation of Mongolian pastures and on increased pressure on pastures; the selection of indicators for the degradation of pastures (the deterioration of the conditions of pastures, the causes of the degradation of pastures; the increase of undesirable plant species).

In 2016, a sociological survey was conducted among 224 families of livestock breeders using the random sampling method. The questionnaire consisted of 54 questions, which were grouped into nine blocks. The questions were related to the nomadic lifestyle, the use of pastures, and methods to improve pastures [68].

Our studies have made it possible to characterize the impact of desertification on households and the health of the local population to identify the main problems of the population’s water supply in the model areas, and to describe the dynamics of the socio-economic status of the population living in the areas affected by desertification.

The majority of the respondents (more than 50.0%) confirm the intensification of desertification processes and its impact on traditional farming, noting that a general decrease in income level from economic activities. However, it should be noted that official statistics, reviews of the socio-economic development of Mongolia, and empirical calculations are more optimistic in comparison with the estimates of the local population about their own financial situation. This suggests that surveyed respondents from households in different parts of Mongolia are adversely affected by increasing desertification.

By analyzing the survey data, we revealed the following proportions of respondents who believe that desertification has an effect on the health of their family members: (1) in soums located in aimags near the Gobi Desert, Gobi aimags, and central aimags—more than half of the respondents; (2) in Chuluunkhoroot soum, located in Dornod aimag in eastern Mongolia—50.0%; (3) in Nogoonnuur and Bukhmurun soums, located in Bayan-Ulgii and Uvs aimags in western Mongolia—14.3% and 33.3%, respectively. Consequently, though desertification has an impact on farming in the north-west of Mongolia, the current situation in that area is generally favorable.

The lack of fresh water and its poor quality threaten the health of people living in the model areas. In view of this, a clean and adequate drinking water supply for the population is a first-priority issue. Additionally, the accessibility of a water supply is a key factor for households in modern conditions. A subsistence farm becomes the main source of family income in conditions where there is a deficiency (or absence) of any earnings. The availability and use of water in Mongolia determine the size of a herd and the area of cultivated land. 

The analysis shows that the existing lack of water and its poor quality, along with low purchase prices for agricultural products, will inevitably lead to a decline in family income. In turn, this would widen the gap between the living standards of rural and urban populations, as well as increase urban migration.

According to the results of the surveys, households are dependent, not only on socio-economic factors, but also on natural ones, which threaten the very existence of a nomadic household to an even greater degree. The low income of the population, combined with limited earning capacity, significantly reduces the population’s living standards. In light of this, desertification is becoming a major destabilizing factor for traditional nomadic households, compelling the population to seek better living conditions in cities. Among the desertification consequences are: the lack of pastures; a reduction in the number of plant species and the quality of grasslands; a reduction in the volume of water for drinking and for domestic and livestock watering purposes; an increase in morbidity, etc.

New forms of economic activity, nonconventional for a nomadic society (in particular, vegetable growing), are not expected to be the main source of a household’s income in the near future. Against this background, the state must step in to regulate the quality and quantity of pastures, along with grazing intensity, and to take responsibility for increasing the living standards of the population, since the traditional principles, on which the management of households in the nomadic society were based, no longer work.

## 5. Conclusions

The main effects of global climate change in Mongolia include an increase in the average temperatures and changes in the regime and type of precipitation, which have a negative impact on the management of nomadic households. The changes in the traditional farming practices of Mongolian households have increased the human impact on natural landscapes. The increase in the total number of livestock (the five-fold increase in the number of goats and the two-fold increase of their shares in the common livestock structure) and the concentration of the population and livestock in the northern and central aimags have become reasons that trigger land conflicts between livestock breeders and crop farmers, and between locals and migrants from other aimags.

The processed results of the sociological survey showed the presence of environmental, social, and economic factors, their cause–effect relationships, and their forms of manifestation.

Our studies have shown that the socio-economic consequences of desertification are very important, and their mitigation determines the living conditions and social well-being of the local population, which is concerned about desertification and its increasing impact on traditional farming. In general, the population’s living standards, the employment possibilities, and the availability of water and pastures in soums located in the north-west part of Mongolia are much better than those in the north-eastern part of the country. However, the traditional lifestyle of nomadic livestock breeders has remained nearly unchanged in the Mongolian areas near the Gobi Desert.

The problem of mitigating the negative socio-economic consequences of increased desertification for nomadic households requires the development of a scientific basis for state policy in the field of social support and the preservation of traditional adaptive nature management in Mongolia.

## Figures and Tables

**Table 1:** Livestock population dynamics in Mongolia, million heads.

Type of Livestock	1990	1995	2000	2005	2010	2015	2018
Horses	2.3	2.7	2.7	2.0	1.9	3.3	3.9
Cattle	2.9	3.3	3.1	2.0	2.2	3.8	4.4
Camels	0.5	0.4	0.3	0.3	0.3	0.4	0.5
Sheep	15.1	13.7	13.9	12.9	14.5	24.9	30.6
Goats	5.1	8.5	10.3	13.3	13.9	23.6	27.1
Total	25.9	28.6	30.3	30.5	32.8	56.0	66.5

Source: [61].

**Table 2:** Main characteristics of the surveys conducted in the model areas in Mongolia ^1^.

Aimag	Soum	Year of Survey	Number of Surveyed Households	Number of Households in Soum ^3^	Sample Proportion, %	Population of Soums, Persons ^3^
Bulgan	Dashinchilen	2009	44	500	8.8	2175
Dundgovi	Saintsagaan	2010	112	1060	10.6	
Darkhan-Uul	Orkhon	2011	79	570	13.9	3140
Umnugovi	Tsogttsetsii ^2^	2012	10	350	2.9	4725
Dornod	Chuluunkhoroot	2018	47	247	19.0	1785
Bayan-Ulgii	Nogoonnuur	2019	7	1550	0.5	7976
Uvs	Bukhmurun	2019	15	496	3.0	2284
Total			314	4773		

^1^ Compiled by the authors, using the data from [18,64]; ^2^ Only a survey of experts was carried out in view of the limitations of the duration of the field study; ^3^ Number of households and population in a soum in previous years was used for calculation of sampling.

**Table 3:** The average monthly income of households in the model areas in comparison with the regional and Mongolian averages (according to national statistics), thousand tugriks ^1^.

Soum	The Average Monthly Income of Households, Thousand Tugriks			Ratios of the Averages (Soum to Region and Soum to Mongolian National), %
	The Average for Soum, According to the Survey	The Regional Average ^4^	The Mongolian National Average ^4^	
Dashinchilen	303.6	309.5	363.6	98.1/83.5
Saintsagaan	295.2	407.0	402.5	72.5/73.3
Orkhon	421.5	476.9	448.0	88.4/94.1
Tsogttsetsii	912.1 ^2^	583.4	573.5	156.3/159.0
Chuluunkhoroot ^3^	–	798.2	1035.5	77.1
Nogoonnuur ^3^	–	1029.8	1181.1	87.2
Bukhmurun ^3^	–	1029.8	1181.1	87.2

^1^ Compiled by the authors, using the data from [18,64]; ^2^ The expert data on the average income of households were used for calculations; ^3^ The questionnaire we used for surveys in 2018–2019 did not include a question on the average monthly income of households. The official statistics of Mongolia do not contain the data on the average monthly income of households, broken down by aimags; ^4^ The regional and the Mongolian national average monthly income of households in previous years was used for the calculation.
